# Increased activation of blood neutrophils after cigarette smoking in young individuals susceptible to COPD

**DOI:** 10.1186/s12931-014-0121-2

**Published:** 2014-10-10

**Authors:** Susan JM Hoonhorst, Wim Timens, Leo Koenderman, Adèle T Lo Tam Loi, Jan-Willem J Lammers, H Marike Boezen, Antoon JM van Oosterhout, Dirkje S Postma, Nick HT ten Hacken

**Affiliations:** Department of Pulmonary Diseases, University of Groningen, University Medical Center Groningen, Groningen, The Netherlands; GRIAC research institute, University of Groningen, University Medical Center Groningen, Groningen, The Netherlands; Department of Pathology, University of Groningen, University Medical Center Groningen, Groningen, The Netherlands; Department of Respiratory Medicine, University Medical Center Utrecht, Utrecht, The Netherlands; Department of Epidemiology, University of Groningen, University Medical Center Groningen, Groningen, The Netherlands; Lab of Allergy and Pulmonary Diseases, University of Groningen, University Medical Center Groningen, Groningen, The Netherlands

**Keywords:** Acute smoking, COPD, Susceptibility, Biomarkers, Inflammation

## Abstract

**Background:**

Cigarette smoking is the most important risk factor for Chronic Obstructive Pulmonary Disease (COPD). Only a subgroup of smokers develops COPD and it is unclear why these individuals are more susceptible to the detrimental effects of cigarette smoking. The risk to develop COPD is known to be higher in individuals with familial aggregation of COPD. This study aimed to investigate if acute systemic and local immune responses to cigarette smoke differentiate between individuals susceptible or non-susceptible to develop COPD, both at young (18-40 years) and old (40-75 years) age.

**Methods:**

All participants smoked three cigarettes in one hour. Changes in inflammatory markers in peripheral blood (at 0 and 3 hours) and in bronchial biopsies (at 0 and 24 hours) were investigated. Acute effects of smoking were analyzed within and between susceptible and non-susceptible individuals, and by multiple regression analysis.

**Results:**

Young susceptible individuals showed significantly higher increases in the expression of FcγRII (CD32) in its active forms (A17 and A27) on neutrophils after smoking (p = 0.016 and 0.028 respectively), independently of age, smoking status and expression of the respective markers at baseline. Smoking had no significant effect on mediators in blood or inflammatory cell counts in bronchial biopsies. In the old group, acute effects of smoking were comparable between healthy controls and COPD patients.

**Conclusions:**

We show for the first time that COPD susceptibility at young age associates with an increased systemic innate immune response to cigarette smoking. This suggests a role of systemic inflammation in the early induction phase of COPD.

**Trial registration:**

Clinicaltrials.gov: NCT00807469

**Electronic supplementary material:**

The online version of this article (doi:10.1186/s12931-014-0121-2) contains supplementary material, which is available to authorized users.

## Background

Cigarette smoking is the most important risk factor for Chronic Obstructive Pulmonary Disease (COPD) [1]. However, only a proportion of all smokers, about 15-20%, will actually develop COPD, the so-called ‘susceptible’ smokers. It is still unclear which factors determine why these individuals are more sensitive to the detrimental effects of cigarette smoking compared with ‘non-susceptible’ smokers.

To better understand how cigarette smoking leads to irreversible lung damage and chronic airflow obstruction, knowledge of the initial responses to cigarette smoking might be very useful. Several studies investigated the acute inflammatory and oxidative stress responses to cigarette smoking in animal and *in vitro* models, yet only a few studies investigated these responses in humans [2]. These studies focused generally on COPD patients and ‘healthy smokers’ without airway obstruction. However, aging and the cumulative amount of pack-years smoking may lead to changes in the airways and lung parenchyma in both groups, likely affecting their response to cigarette smoking. Particularly in COPD, the structural changes in the lung may lead to a different response to smoking. For this reason, it might be hypothesized that the very first responses to cigarette smoking in healthy young individuals with a low number of pack-years is an ideal model to investigate the induction and early progression towards COPD.

Several family studies have provided evidence that a genetic predisposition is involved in the smoking-related development of COPD. Silverman et al. showed that smoking or ex-smoking in first degree relatives of early-onset COPD probands associates significantly with lower forced expiratory volume in one second (FEV_1_) values compared to relatives of control subjects [3]. Several other studies have demonstrated that the combination of smoking and familial clustering of COPD strongly associates with a higher risk for COPD [4-6]. Although a history of familial COPD may help to identify smokers who are susceptible to develop COPD themselves, a more discriminative biomarker would be welcome in the field of preventive medicine. Additionally, elucidating the smoking-induced pathogenesis of COPD in susceptible individuals may ultimately lead to the identification of new drug targets.

The aim of this study was to identify early biomarkers of COPD susceptibility by investigating acute responses to cigarette smoke in young (18-40 years) individuals susceptible and non-susceptible to develop COPD, based on a high prevalence or absence of COPD in smoking relatives. All subjects smoked three cigarettes in one hour. Before and after smoking, inflammatory markers were determined in peripheral blood and bronchial biopsies. We hypothesized that susceptible individuals exhibit a different systemic and local inflammatory response compared to non-susceptible individuals. In addition, we investigated the acute response to cigarette smoking in older (ex) smokers with and without COPD, to assess if responses to cigarette smoking change after many years of smoking.

## Methods

### Study population

Young individuals (age 18-40 years) who are susceptible or non-susceptible to develop COPD were included [7]. All young subjects were intermittent smokers, able to quit smoking for at least 2 days and start smoking on request. Furthermore, we included mild-to-moderate COPD patients (FEV_1_ 30-80% predicted, FEV_1_/FVC <0.7, >10 pack-years), and smokers without airway obstruction (FEV_1_/FVC >0.7, >20 pack-years). Exclusion criteria are mentioned in the online supplement (Additional file 1).

The study was performed at the University Medical Center Groningen (UMCG) (NCT00807469, http://clinicaltrials.gov/show/NCT00807469). The medical ethics committee of the UMCG approved the study protocol and all subjects gave written informed consent.

### Study design

**Figure 1:**
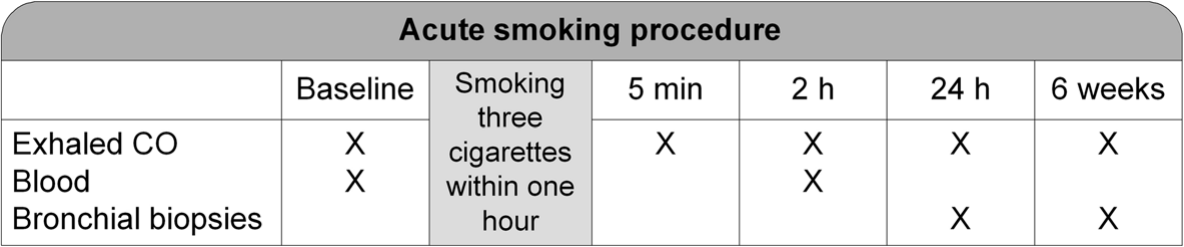
**Time frame of the acute smoking procedure.** *Definition of abbreviations:* CO = carbon monoxide, min = minutes, h = hours. Exhaled CO was obtained at baseline, directly after smoking, and 2 hours after smoking the last cigarette. Blood samples were collected at baseline and 2 hours after smoking the last cigarette. Bronchial biopsies were obtained 24 hours after smoking. Six weeks later bronchial biopsies were obtained as baseline measurement. Subjects refrained from smoking during two days before the baseline measurements and the baseline bronchoscopy after 6 weeks. In addition, subjects refrained from smoking after the acute smoking procedure until the 24 hrs bronchoscopy.

Baseline and follow-up measurements were performed after smoking three cigarettes within one hour (Figure 1). Subjects quitted smoking for at least two days prior baseline visits, and refrained from smoking between the acute smoking procedure and the 24-hrs bronchoscopy. Refraining from smoking was verified by exhaled carbon monoxide (CO) measurements being <5 parts per million (ppm) and sufficient inhalation of the three cigarettes by a rise in CO (Micro^+^ Smokerlyzer®, Bedfont Scientific Ltd, Kent, England). Subjects were not allowed to participate in the acute smoking procedure if their CO measurement was >5 ppm at baseline.

### Measurements

Demographic characteristics were obtained and spirometry, body plethysmography and CO-diffusion were performed according to standardized guidelines [8,9].

Before and after smoking, blood was collected in sodium heparin tubes or serum tubes to perform flow cytometry analysis (FACs) on neutrophil activation markers and cytokine quantification respectively. Detailed methods are described in the online supplement (Additional file 1). Briefly, leucocytes were triple stained with antibodies against (FcγRII) CD32, Mac-1 (CD11b), ICAM-1 (CD54), IL-8 receptors (CD181/CXCR1, CD182/CXCR2) combined with antibodies directed against L-selectin (CD62L) and FcγRIII (CD16). Additionally, the expression of the active form of FcγRII (CD32) was identified by monoclonal phages antibodies MoPhab A17 and A27 [10]. Cells were analyzed in a flow cytometer (FACScalibur; BD Biosciences). Within the granulocyte population (identified based on forward (FCS) and side-scatter (SSC)), neutrophils were identified by CD16^high^ expression and eosinophils by CD16^low^ expression. Flow cytometry data was analysed by FCS Express Version 3 (De Novo software) and median fluorescence intensities (MFI) were calculated. Cytokine quantification was performed by multiplex analyses (Milliplex, Millipore Corporation, Billerica, MA, USA).

Bronchial biopsies were taken from subsegmental carinae of the right or left lower lobe. Briefly, biopsies were fixed in 4% neutral buffered formalin, processed and embedded in paraffin and cut in 3 μm sections. Immunohistochemical stainings were performed using the DAKO autostainer (DAKO, Glostrup, Denmark) using antibodies against inflammatory cells. Detailed immunohistochemistry and quantification procedures are presented in the online supplement (Additional file 1).

### Data analyses

Group characteristics were analyzed using Mann-Whitney U tests or Chi-squared tests. The Wilcoxon signed-rank test was performed to test acute smoking effects within groups. Absolute changes with smoking were analyzed between groups using Mann-Whitney U tests. Multiple linear regression analysis was performed with absolute change in the variables tested as dependent variable and susceptibility to COPD (n/y) as predictor variable. Models were adjusted for relevant co-variables. Data were normalized by log-transformation if necessary. Linear regression models were considered valid if the residuals were normally distributed. Statistical analyses were performed using the statistical program IBM SPSS Statistics version 20.

## Results

### Subjects

Table 1 presents the clinical characteristics of subjects that were included in the study: 50 young individuals, 29 non-susceptible and 21 susceptible, and 40 older subjects, 27 healthy controls and 13 COPD patients. All subjects successfully performed the acute smoking procedure. However, from the total group (n = 90) 6 subjects had missing data in the flow cytometry analyses due to technical reasons and 19 subjects (young non-susceptible: n = 4, young susceptible: n = 7, healthy controls: n = 7, COPD patients: n = 1) had incomplete bronchial biopsy data because subjects did not want to undergo a second bronchoscopy.

**Table 1:** **Group characteristics**

	**Young (<40 years)**		**Old (>40 years)**	
	**Non-susceptible**	**Susceptible**	**Healthy controls**	**COPD**
	***(n = 29)***	***(n = 21)***	***(n = 27)***	***(n = 13)***
Age, years	21 (20-23)	31 (22-38)*	51 (46-62)	66 (64-70)^†^
Gender, male n (%)	17 (59)	11 (52)	23 (85)	13 (100)
Pack-years	1 (0-3)	5 (2-10)*	26 (23-36)	32 (23-46)
Current smokers, n (%)	29 (100)	13 (62)*	26 (96)	10 (77)
Ex-smokers, n (%)	0 (0)	0 (0)	1 (4)	3 (23)
Non-smoker, n (%)	0 (0)	8 (38)	0 (0)	0 (0)
Cig./day for smoking subjects, n	3 (1-10)	8 (2-17)	14 (8-20)	6 (3-14)^†^
FEV_1_, %predicted	106 (101-112)	110 (104-114)	106 (102-116)	65 (60-75)^†^
FEV_1_/FVC, %	85 (83-91)	81 (78-87)*	78 (74-83)	50 (38-59)^†^
RV/TLC, %	22 (19-24)	25 (23-28)	32 (28-37)	39 (34-48)^†^
TLCO/VA, %predicted	100 (92-110)	95 (82-105)	100 (91-106)	75 (63-96)^†^
MEF_50_, %predicted	97 (85-119)	94 (85-108)	90 (80-151)	23 (12-29)^†^
hsCRP, mg/L	0.7 (1.6-1.9)	1.0 (0.6-2.2)	1.9 (0.6-3.8)	2.9 (1.0-5.0)
Blood neutrophils, ×10^9^/L	3.3 (2.7-3.9)	3.8 (2.9-4.4)	3.5 (2.7-4.7)	3.8 (3.3-5.0)
Blood eosinophils, ×10^9^/L	0.16 (0.13-0.26)	0.12 (0.10-0.19)	0.17 (0.10-0.20)	0.20 (0.1-0.4)

*Definition of abbreviations:* *n* number, *FEV*_*1*_ Forced Expiratory Volume in one second, *FVC* Forced Vital Capacity, *RV* Residual Volume, *TLC* Total Lung Capacity, *TLCO/VA* transfer coefficient for carbon monoxide, *MEF*_*50*_ maximal expiratory flow at 50% of vital capacity, *hsCRP* high-sensitivity C-Reactive Protein.

Data are expressed as medians with interquartile ranges (IQR), unless stated otherwise. *p-value <0.05, young susceptible versus young non-susceptible. ^†^p-value <0.05, COPD versus healthy controls.

### Exhaled CO

The baseline median (IQR) exhaled CO value was 1 (1-2) in the whole group, and values were significantly increased in all groups after smoking; 5 (3-10) in young non-susceptible, 7 (4-11) in young susceptible, 8 (6-10) in old healthy controls and 5 (3-8) in COPD patients respectively. There were no significant differences between the study groups in median exhaled CO levels after smoking.

### Flow cytometry on systemic inflammatory cells

**Figure 2:**
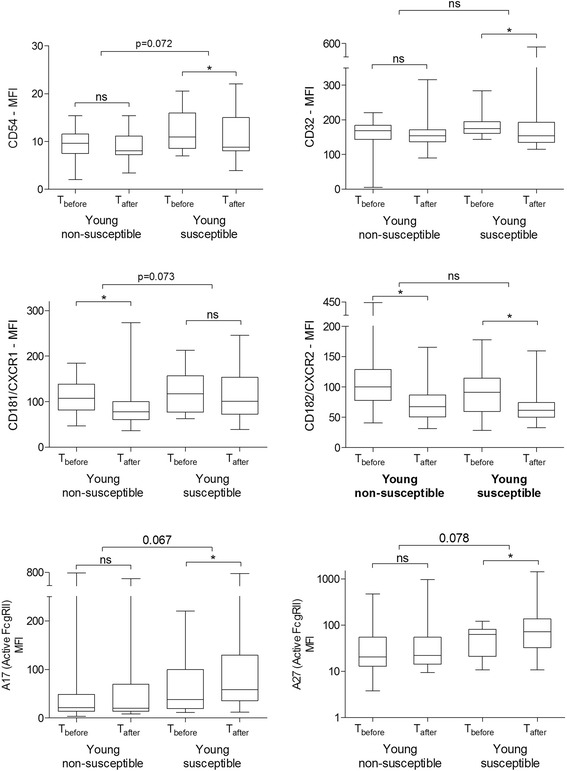
**Effects of acute smoking on expression of neutrophil activation markers in young subjects.** Values are expressed as median fluorescence intensity (MFI) with range, before and two hours after smoking. The responses to cigarette smoke within groups were analyzed by Wilcoxon signed-rank tests. Differences in responses to cigarette smoke between susceptible and non-susceptible groups were analyzed by comparing delta’s (T_after_-T_before_) using Whitney U tests. *p < 0.05, NS = not significant.

**Figure 3:**
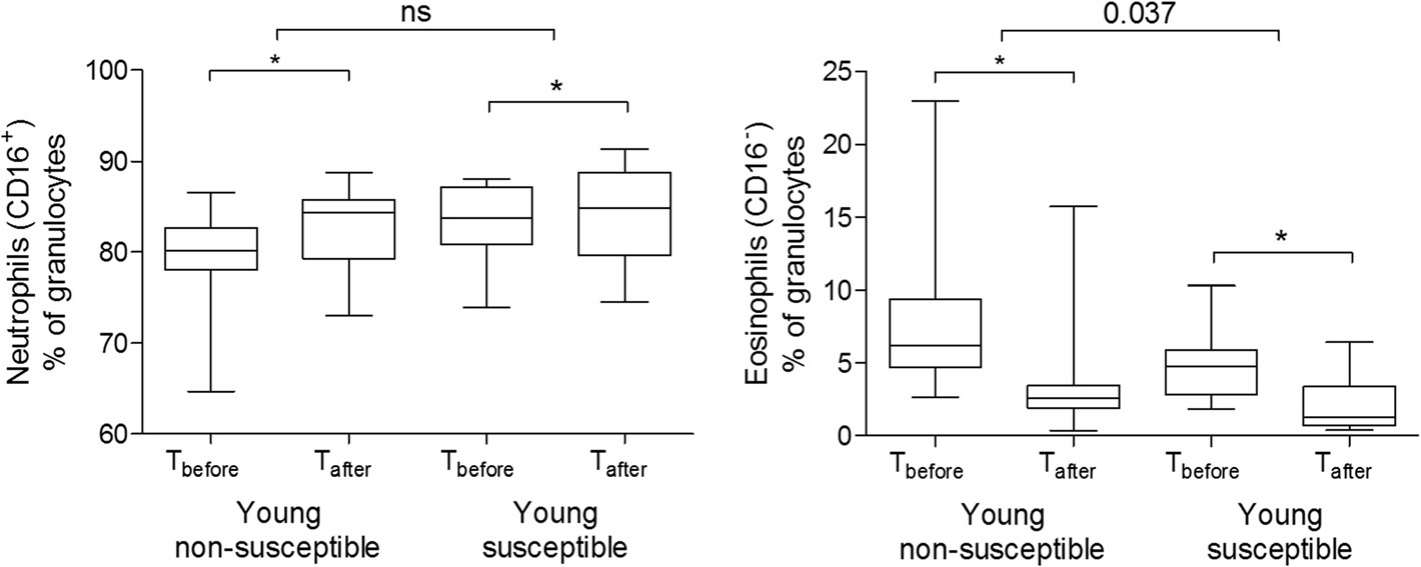
**Effects of acute smoking on total neutrophils and eosinophils in young subjects.** Values are expressed as median percentage with range, before and two hours after smoking. The responses to cigarette smoke within groups were analyzed by Wilcoxon signed-rank tests. Differences in responses to cigarette smoke between susceptible and non-susceptible groups were analyzed by comparing delta’s (T_after_-T_before_) using Whitney U tests. *p < 0.05, NS = not significant.

Table 2 presents changes in cell-surface marker expression on neutrophils in the young groups with smoking. Absolute values before and after smoking are presented in Additional file 1: Table E1. In the susceptible group, CD32 and CD54 expression decreased, and expression of active FcγRII (clones A17 and A27) increased significantly as demonstrated in Figure 2. In the non-susceptible group CD181/CXCR1 expression was significantly decreased. Figures 2 and 3 show that CD182/CXCR2 expression, and percentages of eosinophils (CD16^−^ granulocytes) and neutrophils (CD16^+^ granulocytes) similarly decreased in the two groups.

**Table 2:** **Neutrophil activation markers measured in blood by flow cytometry 2 hours after smoking**

	**Change with smoking**		
	**Young non-susceptible**	**Young susceptible**	***p-value*** ^**†**^
	***(n = 27)***	***(n = 20)***	
CD16^+^ Neutrophils	3.4 (-1.3;6.7)*	1.8 (0.2;4.8)*	NS
CD16^−^ Eosinophils	−3.6 (-5.6;-2.4)*	−2.3 (-3.9;-1.3)*	0.037
CD11b (Mac-1)	−4.6 (-20.3;18.6)	9.9 (-16.4;76.6)	NS
CD32 (FcγRII)	−12.6 (-23.9;2.67)	−12.3 (-31.7;-6.1)*	NS
CD54 (ICAM-1)	−0.8 (-1.6;0.48)	−1.6 (-23.0;-0.13)*	NS (0.072)
CD181/CXCR1 (IL-8 receptor)	−19.2 (-48.2;-2.6)*	−5.2 (-27.9;9.8)	NS (0.073)
CD182/CXCR2 (IL-8 receptor)	−23.5 (-53.0;-6.5)*	−27.7 (-46.5;0.7)*	NS
A17 (active FcγRII)	3.26 (-3.0;3.3)	14.8 (2.6;71.0)*	NS (0.067)
A27 (active FcγRII)	0.4 (-9.6;14.6)	19.0 (0.8;67.8)*	NS (0.078)

Values are expressed as median change (T_after_-T_before_) in fluorescence intensity (MFI) with interquartile ranges (IQR), two hours after smoking. *Significant response to cigarette smoke within the group (Wilcoxon signed-rank tests, p < 0.05). ^†^p-values for differences in responses to cigarette smoke between susceptible and non-susceptible subjects (Mann-Whitney U tests, NS = not significant).

Differential responses to smoking were borderline significant between groups as follows: CD54 decreased more and A17 and A27 increased more in the young susceptible group (Figure 2). CD181/CXCRI expression increased more in the young non-susceptible group (Figure 2). Finally, eosinophil percentages decreased more in the non-susceptible group (Figure 3).

### Cytokine concentrations in serum

Changes in cytokine levels with smoking are presented in Table 3 (see Additional file 1: Table E1 for absolute values before and after smoking). IL-8 and GM-CSF were significantly decreased in the non-susceptible group, whereas IL-7 significantly increased. Furthermore, TNFα was significantly decreased in both groups. The changes with smoking were not significantly different between the susceptibility groups.

**Table 3:** **Cytokines measured in blood 2 hours after smoking**

	**Change with smoking**		
	**Young non-susceptible**	**Young susceptible**	***p-value*** ^**†**^
	***(n = 29)***	***(n = 21)***	
IL−1β	0.00 (0.00;0.00)	0.00 (0.00;0.00)	NS
IL−6	0.00 (−0.25;0.15)	−0.04 (−1.0;0.06)	NS
IL−8	−0.70 (−1.56;−0.01)*	−0.24 (−1.03;0.68)	NS
GM−CSF	0.00 (−0.20;0.00)*	0.00 (−0.54;0.00)	NS
TNFα	−0.27 (−0.89;0.00)*	−0.35 (−1.11;0.16)*	NS
IFNγ	0.00 (−0.84;1.10)	−0.49 (−1.59;1.13)	NS
IL-2	0.00 (−0.46;0.70)	0.00 (−0.70;0.48)	NS
IL-4	0.00 (−0.91;0.00)	0.00 (−0.11;0.00)	NS
IL-5	0.00 (−0.09;0.09)	0.00 (−0.05;0.00)	NS
IL-7	0.79 (0.00;3.97)*	0.00 (−1.07;3.39)	NS
IL-10	0.00 (−1.81;0.00)	0.00 (−1.35;1.46)	NS
IL-12p70	0.00 (−0.31;0.24)	0.00 (−0.83;0.31)	NS
IL-13	0.00 (0.00;1.65)	0.00 (−1.10;1.63)	NS

Values are expressed as median change (T_after_-T_before_) in cytokine concentration (pg/ml) with interquartile ranges (IQR), two hours after smoking. *Significant response to cigarette smoke within the group (Wilcoxon signed-rank tests, p < 0.05). ^†^p−values for differences in responses to cigarette smoke between susceptible and non−susceptible subjects (Mann−Whitney U tests, NS = not significant).

### Inflammatory cells in bronchial biopsies after smoking

Table 4 presents the changes in inflammatory cell counts in bronchial biopsies 24 hours after smoking (see Additional file 1: Table E1 for absolute values before and after smoking). Eosinophils (EPX immunopositivity) was significantly increased in the non-susceptible group. The changes of bronchial cell counts were not significantly different with smoking between groups.

**Table 4:** **Inflammatory cells in bronchial biopsies 24 hours after smoking**

	**Change with smoking**		
	**Young non-susceptible**	**Young susceptible**	***p-value*** ^**†**^
	***(n = 25)***	***(n = 14)***	
*Submucosal*			
CD3^+^ T-cells	6.8 (−21.9;21.6)	3.2 (−9.1;15.3)	NS
CD4^+^ T-cells	0.0 (−2.8;11.8)	−0.4 (−7.4;1.5)	NS
CD8^+^ T-cells	0.2 (−0.8;16.5)	−8.9 (−29.9;11.3)	NS
FOXP3^+^ T-cells	0.9 (−1.9;2.2)	0.2 (−0.3;1.8)	NS
CD68^+^ macrophages	2.3 (−1.2;7.2)	−0.4 (−4.0;2.6)	NS
AA1^+^ mast cells	0.0 (−2.1;11.8)	1.1 (−3.5;2.7)	NS
EPX^+^ eosinophils	0.8 (0.0;1.0)*	0.0 (−0.7;1.0)	NS
NP57^+^ neutrophils	1.2 (−5.8;5.2)	1.7 (−6.8;7.4)	NS
% E-selectin pos. vessels	0.0 (−1.0;0.0)	0.0 (−5.2;0.0)	NS

Values are expressed as median change (T_after_-T_before_) in cell counts with interquartile ranges (IQR), 24 hours after smoking. Inflammatory cells are expressed as cell counts/0.1 mm^2^. *Significant response to cigarette smoke within the group (Wilcoxon signed-rank tests, p < 0.05). ^†^p-values for differences in responses to cigarette smoke between susceptible and non-susceptible subjects (Mann-Whitney U tests, NS = not significant).

### Susceptibility as predictor of systemic responses to cigarette smoking

We assessed whether susceptibility predicted the changes in expression of CD54, CD181/CXCR1, active FcγRII (clones A17 and A27) and percentage of eosinophils with smoking by multiple linear regression models. Table 5 shows that susceptibility was a significant predictor of the change in A17 and A27 expression after smoking, independently of expression at baseline, age and smoking status. Susceptibility was not associated with CD54 and CD181/CXCR1 expression, and percentage of eosinophils.

**Table 5:** **Associations of susceptibility (no/yes) with the change in expression of neutrophil markers after smoking**

**Dependent variable: change in expression with smoking**	**Predictor variable: susceptibility y/n** ^**†**^		
***n = 47***	**B**	**S.E.**	**p-value**
(CD181/CXCR1 (IL-8 receptor)^‡^	0.130	0.106	0.227
CD54 (ICAM-1)	−1.043	0.878	0.241
A17 (active FcγRII)^‡^	0.127	0.051	0.016*
A27 (active FcγRII)^‡^	0.102	0.045	0.028*
CD16^−^ Eosinophils	0.680	0.695	0.334

Different multiple regression models with susceptibility to COPD (y/n) as predictor value and change in expression of neutrophil markers (CD181/CXCR1, CD54, A17 or A27) or % eosinophils after smoking (T_after_-T_before_) as dependent variable. B = regression coefficient. *Significant (p < 0.05). ^†^all models were adjusted for expression of marker at baseline, age and current smoking n/y. ^‡^Data were log-transformed.

**Figure 4:**
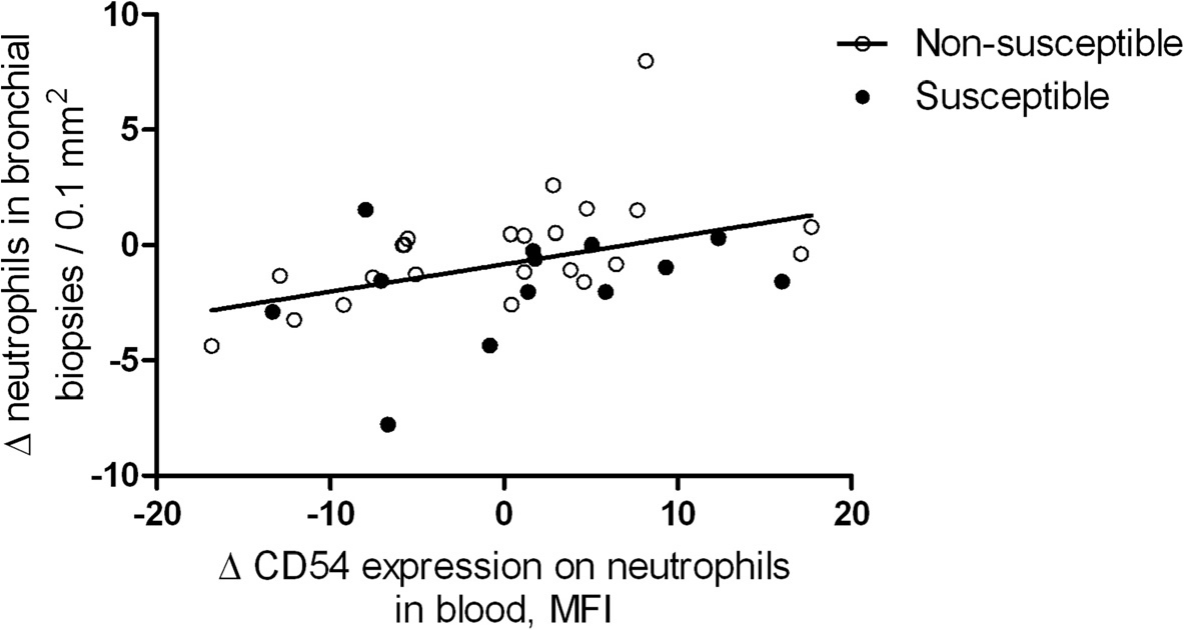
**Association between change in expression of neutrophil activation markers and change in number of bronchial NP57** ^**+**^ **neutrophils in bronchial biopsies after smoking.** Values are expressed as change in median fluorescence intensity (MFI) (T_before_ – T_after_), two hours after smoking.

Additionally, using multiple linear regression analysis, we investigated if the changes in expression of neutrophil activation markers were predictors of the change in number of neutrophils in bronchial biopsies. Both a higher increase in CD54 expression of blood neutrophils and susceptibility were significant predictors of a higher increase of bronchial neutrophils counts (Table 6, Figure 4).

**Table 6:** **Association of change in expression of neutrophil activation markers and susceptibility with change in number of bronchial NP57** ^**+**^ **neutrophils in bronchial biopsies**

**Outcome variable: change in number of bronchial NP57** ^**+**^ **neutrophils with smoking**		
***n = 39*** **, R** ^**2**^ **= 0.620**	***β***	***p-value***
Change in CD54 expression (T_after_-T_before_)	0.271	0.044*
NP57 expression at baseline	−0.597	<0.001*
Susceptibility, n/y	0.386	0.013*
Current smoking, n/y	0.309	0.037*

Multiple regression model with susceptibility to COPD (y/n) as predictor value and change in expression of neutrophil count in bronchial biopsies after smoking (T_after_-T_before_) as dependent variable. *Β* = standardized regression coefficient. *Significant (p < 0.05).

### Effects of acute smoking in COPD patients and smokers without airway obstruction

Data of the acute smoking procedure in the old groups are presented in Additional file 1: Table E2. Briefly, we observed no significant differences in the change of neutrophil marker expression after smoking between COPD patients and controls, whereas the decrease in the percentage of eosinophils was larger in COPD patients. IL-6 and IL-8 levels in blood decreased after smoking in healthy controls, a change that was close to significance when compared with the change in COPD patients. Finally, the change in number of bronchial neutrophils significantly differed between groups, showing an increase in healthy controls and a decrease in COPD patients.

## Discussion

This is the first human study using an acute smoking design in a population of young and old individuals being susceptible or non-susceptible to develop COPD. The focus of this study was on the comparison of the acute response to cigarette smoking in young individuals, older subjects were investigated to assess if responses change after many years of smoking. We demonstrated that susceptibility to develop COPD at young age associates with an enhanced innate immune response to cigarette smoking in peripheral blood when compared with non-susceptible individuals, suggesting that a systemic inflammatory component is involved during the induction of COPD.

Our most important finding is that peripheral blood neutrophil activation markers were differentially expressed after smoking between young susceptible and young non-susceptible subjects. Previous human studies have shown that the number of peripheral blood neutrophils increases after acute smoking [2], a finding that we confirmed, i.e. neutrophils (CD16^+^ granulocytes) significantly increased in both the susceptible and non-susceptible group (Figure 3). However, the activation of neutrophils is a well-described multi-step process, generally starting with priming (pre-activation) caused by chemotaxins or cytokines, leading to upregulation of integrins and adhesion molecules (e.g. CD11b, ICAM-1) [11,12]. Additionally, primed neutrophils can be recognized by MoPhab antibodies A17 and A27 since they bind FcγRII (CD32) only in the context of primed cells and exquisitely capable to detect primed cells in the circulation [10,13]. We found that acute smoking significantly increased median A17 and A27 expression only in the group of young susceptible subjects (Table 2, Figure 2). This effect was further confirmed by regression analyses, showing that this increase was independently of age, smoking status and marker expression at baseline. In contrast, receptors involved in adhesion and migration tended to decrease after smoking, which was significant for ICAM-1 and CD182/CXCR2 markers in susceptible subjects and for CD181/CXCR1 and CD182/CXCR2 in non-susceptible subjects. Taken together these data suggest that circulating neutrophils become more activated immediately after smoking, and particularly so in young susceptible subjects.

The underlying mechanisms are complex. Some *in vitro* studies have shown that circulating neutrophils of smokers are pre-activated or primed compared with never-smokers and have a higher capacity to migrate towards chemotactic stimuli, or are more responsive to activating agents [14,15]. We did not investigate chemotactic characteristics of neutrophils, however, our data are pointing at a mechanism by which neutrophils are more easily primed in young susceptible individuals. This may contribute to a higher influx of neutrophils into the airways, leading to more intense inflammation and tissue damage. The trend we found in reduced expression of ICAM-1 in young susceptible individuals supports this hypothesis. Neutrophils with upregulated expression of adhesion molecules may already have left the circulation infiltrating the lung tissue. This concept has also been proposed for eosinophils in allergic asthma by Johansson et al [16]. Interestingly, we demonstrated that a higher increase of ICAM-1 expression on circulating neutrophils upon smoking was associated with a higher increase of bronchial biopsy neutrophils. In the same model, being susceptible to develop COPD and current smoking were independent predictors of neutrophil influx after smoking, indicating that the influx of cells is higher in susceptible individuals who smoke. However, no significant associations were found between bronchial cell counts and the other neutrophil activation markers. This lack in association may be due to the fact that blood and bronchial biopsies were collected at different time points. Nevertheless, it is encouraging that our methods identified subtle alterations in the activation state of circulating neutrophils associated with changes in neutrophil numbers in the airways.

Blood eosinophil numbers decreased after smoking both in young susceptible and non-susceptible subjects, a finding in accordance with our previous study on acute smoking effects in intermittent smokers [17]. Another study in four young healthy women demonstrated a decreased number of eosinophils two hours after smoking of 12 cigarettes [18]. Interestingly, eosinophil numbers also significantly decreased after smoking in COPD patients and healthy controls. The underlying mechanism is yet to be defined, but a similar situation is found upon systemic LPS challenge in man [19]. Apparently, eosinophil homing signals are generated by innate immune signals such as DAMPs (acute smoking) and PAMPs (LPS). Our study did not show associations between smoking-induced changes in eosinophil numbers and cytokine concentrations in blood, suggesting that remaining eosinophils were not responsive to the cytokines with respect to homing of the cells. Another explanation might be that eosinophils migrated from the circulation into the lung tissue. However, we did not find an associated rise in eosinophil numbers in bronchial biopsies after smoking. A final explanation is that toxic substances in cigarette smoke cause apoptosis [20], a phenomenon we did not investigate specifically. Interestingly, susceptible subjects had a deeper fall in eosinophils than non-susceptible subjects in both the young and old population, although this finding did not remain significant in multiple regression analysis. Apparently, the eosinophilic response to cigarette smoke is not contributing to susceptibility, in contrast to the neutrophilic response.

Next we investigated whether the differences in responses to cigarette smoke between young susceptible and non-susceptible subjects were also present between COPD patients and healthy controls. Here we found no differences in expression of neutrophil activation markers in peripheral blood after smoking, which may be due to the fact that they were older and had more pack-years. It is known that age and prolonged smoking increases systemic inflammation [21]. Further, several studies have demonstrated that neutrophils are more activated in COPD patients [22], and this may have obscured a relatively subtle response on recent smoking exposure. However, basal levels of expression markers in both our old groups did not differ, thus a different explanation is required. There, we postulate that the inflammatory response to cigarette smoking after long-term smoking has faded out or has been switched into a more persistent inflammatory response, minimizing the ability to detect subtle changes in neutrophil activation.

Smoking of three cigarettes did not affect inflammatory cell counts in bronchial biopsies 24 hours later. This contrasts with findings in animal models, where acute smoking results in an influx of inflammatory cells in lung tissue 6-24 hours later [23-25]. The time point of 24 hours after smoking was chosen based on animal studies given the lack of data in men [2,7]. It may well be that the response to cigarette smoking in human occurs early after smoking, or that animals were exposed to relatively much higher levels of cigarette smoke. Smoking in human has been shown to increase neutrophils in sputum [17], bronchoalveolar lavage fluid (BALF) [26] and lung tissue using nuclear imaging techniques [27]. Possibly, the main effects of smoking do not take place in the large airways, but at other lung regions like peripheral airways and lung parenchyma. Clearly, our negative biopsy findings can be explained in a number of ways: collecting biopsies too late after smoking, smoking of too few cigarettes, or investigating the wrong lung compartment. Future human studies must take these considerations into account.

The strengths of our study are that we investigated young individuals with normal lung function who are either susceptible or not susceptible to develop COPD and we used a disease-specific challenge to find biomarkers of COPD susceptibility. There are some limitations as well. First, we defined COPD susceptibility on familial history of COPD only; no lung function measurements were performed to verify COPD in family members. However, family history of COPD is a strong risk factor of COPD [5] and we maintained a strict inclusion algorithm [7]. Second, we used exhaled CO to verify smoking abstinence before the acute smoking procedure, yet this is only reliable within 6 hours of smoking cessation. Third, the young susceptible group smoked a higher number of pack-years compared with the susceptible group. Fourth, the number of participants was relatively low, especially in the young susceptible and the COPD group. Additionally, we lost some data because 21% of the subjects did not complete the two bronchoscopies. However, our significant findings are relevant as they were found in spite of the low sample size of this study.

In conclusion, we found that COPD susceptibility at young age associates with an increased activation of peripheral neutrophils after cigarette smoking. This increased innate immune response was not found at old age, likely because the inflammatory response to cigarette smoking has faded out or has been switched into a more persistent inflammatory response as a result of long-term smoking or aging. Our data emphasizes that systemic inflammation contributes likely to the early induction phase of COPD.

## Additional file

Additional file 1:
**Online data supplement.**

## Abbreviations

COPD: Chronic obstructive pulmonary disease
FEV_1_: Forced expiratory volume in one second
FVC: Forced vital capacity
CO: Carbon monoxide
MEF: Maximal expiratory flow
MFI: Median fluorescence intensity
RV: Residual volume
TLC: Total Lung capacity
TLCO: Transfer coefficient for carbon monoxide
FRC: Functional residual capacity
