# Supplementary material for: Increased activation of blood neutrophils after cigarette smoking in young individuals susceptible to COPD
Source: Respir Res. 2014 Oct 10;15(1):121. doi: 10.1186/s12931-014-0121-2 (PMC4203909; doi:10.1186/s12931-014-0121-2)
Supplement: Additional file 1: — Online data supplement. [file 12931_2014_121_MOESM1_ESM.docx]

**Increased activation of blood neutrophils after cigarette smoking**

**in young individuals susceptible to COPD**

Susan J.M. Hoonhorst, Wim Timens, Leo Koenderman, Jan Willem J. Lammers, H. Marike Boezen, Antoon J.M. van Oosterhout, Dirkje S. Postma, Nick H.T. ten Hacken

**Online data supplement**

**METHODS**

**Exclusion criteria**

Exclusion criteria were α-1-antitrypsin deficiency, acute pulmonary infections, prior history of significant inflammatory lung disease other than COPD, active infections, treatment with antibiotics or corticosteroids within 8 weeks, and recent diagnosis of cancer. In addition, medication which could affect the results of the study was excluded, such as anti-inflammatory drugs and immunosuppressive agents.

**Flow cytometry analysis of neutrophilic surface receptors in blood**

Before and after smoking, peripheral blood was collected in tubes containing sodium heparin. Red blood cells were lysed and washed with 5 ml cold PBS + 10g/l albumin (Albuman) + 0.32% sodium citrate. 25 µl of leukocytes (5x10^6^ cells/ml) were triple stained with antibodies to identify the expression of activation markers on neutrophils; (FcγRII) CD32, Mac-1 (CD11b), ICAM-1 (CD54), IL-8 receptors (CD181/CXCR1, CD182/CXCR2) combined with antibodies directed against L-selectin (CD62L) and FcγRIII (CD16) (to identify neutrophil and eosinophil populations, respectively). Cells were analyzed in a flow cytometer (FACScalibur; BD Biosciences). Leucocyte populations were identified based on forward (FCS) and side-scatter (SSC) characteristics. Neutrophils were identified by CD62L^high^ cells and eosinophils by CD16^low^ cells in the granulocytes population.

To identify the active form of FcγRII (CD32) on the surface of granulocytes directly FITC-labelled monoclonal phages antibodies (MoPhab) A17 and A27 were used as described previously (1). In short, 50 µl of whole blood was primed during 5 minutes by 37 °C. After 5 minutes, samples were incubated with antibodies A17, A27, and CD11b during 1 hour. Red blood cells were lysed and analysed in a flow cytometer (FACSCalibur; BD Biosciences). The neutrophil population was identified based on FCS and SSC characteristics.

Antibodies specifications: CD16 (clone 3G), CD62L (clone DREG56) and CD32 (clone FLI8.26) were obtained from BD Pharmingen (San Diego, CA, USA); CD11b (clone 2LPM19c) from DAKO (Copenhagen, Denmark); CD54 (clone MEM-111) from Caltag (San Francisco, CA, USA); CD181/CXCR1 (clone 42705) and CD182/CXCR2 (clone 48311) from R&D systems (Europe, UK).

All data of flow cytometry were analysed by FCS Express Version 3 (De Novo software) and for each antibody the median fluorescence intensity (MFI) was calculated.

**Multiplex analysis**

Before and after smoking, blood was collected in serum tubes containing clot activator and gel for serum separation. The tubes were stored at room temperature during 2-3 hours and centrifugated afterwards (2000 rcfmax at room temperature, 10 minutes). Serum was obtained and stored at -80°C until analyses were performed. Cytokine quantification was performed by multiplex analyses (Milliplex, Millipore Corporation, Billerica, MA, USA).

**Inflammatory cells in bronchial biopsies**

After administering of local anaesthesia (lidocain 2-4%), a flexible bronchoscope was introduced and bronchial biopsies were taken from subsegmental carinae of the right lower lobe. Biopsies were fixed in 4% neutral buffered formalin, processed and embedded in paraffin and cut in 3 µm sections. Quality of biopsies was verified by hematoxylin and eosin (HE) staining. Immunohistochemical stainings were performed using the DAKO autostainer (DAKO, Glostrup, Denmark), by antibodies against T-lymphocytes (CD3 (DAKO), CD4 (Novocastra), CD8 (DAKO)), Treg-lymphocytes (FoxP3, Abcam), neutrophils (NP57, DAKO), macrophages (CD68, DAKO), mast cells (AA1, DAKO), and eosinophils (EPX, Lee Labs). Sections were deparaffinized, antigens were retrieved and then sections were incubated with primary antibodies. Primary antibodies CD3, CD4, FoxP3, NP57, CD68 and AA1 were detected with NovaRed kit (Vector Labs, Burlingame, USA). CD8 was detected using biotinylated anti-mouse IgGI and streptavidin labelled peroxidase antibodies followed y the chromogen Nova red. EPX was detected using biotiniylated anti-mouse and streptavidin alkalin phosphase antibodies followed by the chrmogen Permant Red. All sections were counterstained with methylgreen (greenblue).

Quantification of all stainings was performed by one blinded observer, using ImageScope (Aperio Technologies, version 11.2.0.780). Quantification was performed on the largest of three biopsy sections. The number of positive stained inflammatory cells (i.e. positively stained nuclei) was scored in the submucosal area, 100 µm under the basement membrane and in a total area of 0.1mm^2^ per biopsy (2). Double positive blood vessels stained for CD31 and E-selectin were also scored in the submucosal area, 100 µm under the basement membrane and in the whole biopsy.

**TABLE E1. VALUES BEFORE AND AFTER ACUTE SMOKING IN YOUNG AND OLD GROUPS**

1. **Neutrophil activation markers measured in blood by flow cytometry 2 hours after smoking**

|  | **Young (<40 years)** | | | | **Old (>40 years)** | | | |
| --- | --- | --- | --- | --- | --- | --- | --- | --- |
|  | **Non-susceptible** | | **Susceptible** | | **Healthy controls** | | **COPD patients** | |
|  | (n=27) | | (n=20) | | (n=24) | | (n=13) | |
|  | *Before* | *After* | *Before* | *After* | *Before* | *After* | *Before* | *After* |
| CD11b | 96 | 88 | 104 | 96 | 122 | 136 | 100 | 84 |
|  | (72-129) | (67-129) | (76-143) | (72-209) | (87-166) | (89-198) | (62-133) | (70-157) |
| CD32 | 169 | 154 | 175 | 154 | 184 | 178 | 178 | 160 |
|  | (143-184) | (133-172) | (161-195) | (135-193) | (165-205) | (165-191) | (165-184) | (143-169) |
| CD54 | 10 | 8 | 12 | 9 | 12 | 11 | 11 | 9 |
|  | (8-12) | (7-11) | (9-16) | (8-16) | (8-17) | (8-17) | (7-13) | (6-12) |
| CD181/CXCR1 | 107 | 78 | 118 | 101 | 124 | 116 | 107 | 87 |
|  | (82-138) | (61-100) | (77-157) | (73-154) | (90-151) | (87-169) | (72-124) | (66-120) |
| CD182/CXCR2 | 100 | 67 | 93 | 63 | 93 | 87 | 72 | 56 |
|  | (78-129) | (50-87) | (63-118) | (52-79) | (63-115) | (58-114) | (56-98) | (51-85) |
| A17 | 21 | 20 | 38 | 58 | 41 | 49 | 39 | 44 |
|  | (14-49) | (14-70) | (19-99) | (36-130) | (27-62) | (32-104) | (17-188) | (27-127) |
| A27 | 21 | 22 | 63 | 71 | 48 | 45 | 35 | 67 |
|  | (13-54) | (14-54) | (21-81) | (33-137) | (34-107) | (34-107) | (22-114) | (36-124) |
| Eosinophils | 6.1 | 2.6 | 4.8 | 1.3 | 4.8 | 2.8 | 6.6 | 3.5 |
|  | (4.7-9.3) | (1.4-3.5) | (2.9-5.9) | (0.7-3.4) | (2.4-7.7) | (1.5-4.5) | (3.3-11.1) | (1.6-5.2) |

Values are expressed as median fluorescence intensity (MFI) with interquartile ranges (IQR)

**B. Cytokines measured in blood 2 hours after smoking**

|  | **Young (<40 years)** | | | | **Old (>40 years)** | | | |
| --- | --- | --- | --- | --- | --- | --- | --- | --- |
|  | **Non-susceptible** | | **Susceptible** | | **Healthy controls** | | **COPD patients** | |
|  | (n=29) | | (n=21) | | (n=27) | | (n=13) | |
|  | Before | After | Before | After | Before | After | Before | After |
| IL-1β | 0.13 | 0.13 | 0.13 | 0.13 | 0.13 | 0.13 | 0.13 | 0.13 |
|  | (0.13-0.13) | (0.13-0.13) | (0.13-0.13) | (0.13-0.13) | (0.13-0.13) | (0.13-0.13) | (0.13-0.13) | (0.13-0.13) |
| IL-6 | 0.94 | 0.87 | 1.62 | 1.51 | 2.26 | 1.77 | 2.25 | 2.41 |
|  | (0.13-1.45) | (0.13-1.38) | (0.13-0.13) | (1.12-2.38) | (1.78-4.60) | (1.40-3.65) | (1.19-4.46) | (1.31-4.70) |
| IL-8 | 6.16 | 5.33 | 6.16 | 5.15 | 6.08 | 5.78 | 8.07 | 7.87 |
|  | (4.91-7.47) | (4.03-6.69) | (4.62-7.70) | (3.86-7.64) | (4.46-9.10) | (3.88-8.02) | (7.2-10.12) | (6.27-10.35) |
| GM-CSF | 0.13 | 0.13 | 0.13 | 0.13 | 0.13 | 0.13 | 0.13 | 0.13 |
|  | (0.13-0.58) | (0.13-0.13) | (0.13-1.01) | (0.13-0.68) | (0.13-0.68) | (0.13-0.13) | (0.13-0.13) | (0.13-0.13) |
| TNFα | 5.43 | 5.68 | 5.61 | 5.37 | 5.68 | 5.06 | 5.95 | 5.57 |
|  | (4.20-7.12) | (4.35-6.71) | (3.88-6.97) | (3.68-5.97) | (4.22-8.00) | (3.92-6.77) | (2.63-9.10) | (3.16-8.75) |
| IFNγ | 3.76 | 4.29 | 7.07 | 6.08 | 3.40 | 4.16 | 2.22 | 2.98 |
|  | (2.80-6.77) | (2.33-6.59) | (1.02-9.53) | (3.46-9.43) | (1.59-9.68) | (1.59-6.48) | (1.44-3.95) | (1.74-6.76) |
| IL-2 | 1.10 | 1.84 | 1.84 | 1.84 | 0.27 | 0.47 | 0.79 | 0.25 |
|  | (0.27-1.30) | (0.27-2.80) | (0.47-4.42) | (0.83-3.76) | (0.13-1.95) | (0.13-1.42) | (0.13-1.44) | (0.15-0.90) |
| IL-4 | 0.35 | 0.35 | 0.13 | 0.13 | 0.13 | 0.13 | 0.13 | 0.13 |
|  | (0.13-4.54) | (0.13-1.72) | (0.13-1.72) | (0.13-1.72) | (0.13-1.72) | (0.13-0.35) | (0.13-4.48) | (0.13-4.54) |
| IL-5 | 0.18 | 0.18 | 0.24 | 0.26 | 0.13 | 0.13 | 0.13 | 0.24 |
|  | (0.13-0.39) | (0.13-0.36) | (0.13-0.49) | (0.13-0.45) | (0.13-0.34) | (0.03-0.24) | (0.13-0.32) | (0.13-0.33) |
| IL-7 | 4.42 | 7.00 | 7.16 | 8.3 | 3.97 | 4.42 | 2.77 | 7.00 |
|  | (2.84-7.17) | (4.27-9.60) | (4.55-12.20) | (4.27-13.22) | (1.22-8.84) | (1.22-10.02) | (1.24-8.41) | (3.98-8.84) |
| IL-10 | 5.24 | 4.54 | 4.25 | 4.77 | 5.24 | 4.25 | 5.24 | 4.08 |
|  | (3.30-8.84) | (2.38-8.94) | (0.13-8.60) | (0.13-12.35) | (0.13-9.85) | (0.13-8.96) | (3.85-9.66) | (0.13-5.92) |
| IL-12p70 | 1.01 | 1.19 | 1.97 | 1.71 | 1.71 | 1.45 | 0.61 | 1.19 |
|  | (0.19-1.87) | (0.25-1.84) | (1.23-2.98) | (0.81-2.92) | (0.80-3.33) | (0.94-2.77) | (0.19-1.22) | (0.25-1.33) |
| IL-13 | 2.21 | 2.57 | 2.57 | 4.13 | 2.53 | 2.21 | 1.00 | 1.00 |
|  | (0.46-4.60) | (1.17-6.60) | (0.84-7.02) | (1.56-6.70) | (0.13-7.60) | (0.13-7.59) | (0.55-2.39) | (0.67-2.21) |

Values are expressed as medians with interquartile ranges (IQR)

**C. Inflammatory cells in bronchial biopsies 24 hours after smoking**

|  | **Young (<40 years)** | | | | **Old (>40 years)** | | | |
| --- | --- | --- | --- | --- | --- | --- | --- | --- |
|  | **Non-susceptible** | | **Susceptible** | | **Healthy controls** | | **COPD patients** | |
|  | (n=25) | | (n=14) | | (n=20) | | (n=12) | |
|  | *Before* | *After* | *Before* | *After* | *Before* | *After* | *Before* | *After* |
| CD3^+^ T-cells | 33.6 | 41.8 | 39 | 46.1 | 23.4 | 22.8 | 14.8 | 18.6 |
|  | (19.6-59.9) | (25.7-56.9) | (26.6-51.8) | (33.1-67.0) | (12.1-36.3) | (11.2-30.0) | (8.9-21.2) | (6.8-31.9) |
| CD4^+^ T-cells | 6.0 | 9.1 | 5.2 | 3.1 | 2,0 | 2,0 | 1.2 | 2.8 |
|  | (0.4-11.5) | (3.2-19.2) | (0.5-16.8) | (0.6-11.6) | (0.0-6.8) | (0.2-10.8) | (0.0-1.9) | (0-5.2) |
| CD8^+^ T-cells | 34.4 | 33.0 | 32.4 | 30.1 | 14.4 | 22.9 | 11.7 | 14,0 |
|  | (9.9-64.8) | (22.4-51.4) | (22.4-55.8) | (9.0-47.5) | (7.6-32.4) | (8.7-43.6) | (3.7-19.8) | (3.8-25.8) |
| FOXP3^+^ T-cells | 0.9 | 1.5 | 1.0 | 1.8 | 0.8 | 1,0 | 0.0 | 0.8 |
|  | (0.0-2.8) | (0.8-3.6) | (0.0-3.6) | (0.8-4.2) | (0.0-1.8) | (0.0-2.5) | (0.0-0.8) | (0.0-3.3) |
| CD68^+^ macrophages | 6.1 | 8 | 5.7 | 5.9 | 3.7 | 4.5 | 4.2 | 1.9 |
|  | (1.4-10.3) | (5.5-13.4) | (2.2-12.1) | (0.9-11.6) | (0.8-10.2) | (2.0-8.6) | (2.5-7.4) | (0.0-4.1) |
| AA1^+^ mast cells | 3.2 | 3.5 | 2.9 | 2.6 | 2.2 | 3.4 | 1.8 | 2.9 |
|  | (1.9-5.8) | (1.1-5.9) | (0.8-6.9) | (0.0-3.8) | (0.7-5.6) | (1.4-5.7) | (0.2-3.5) | (0.4-3.0) |
| EPX^+^ eosinophils | 0.0 | 0.8 | 0 | 0 | 0.0 | 0.4 | 0.4 | 0,0 |
|  | (0.0-0.9) | (0.0-1.3) | (0-1.0) | (0.0-1.4) | (0.0-0.5) | (0.0-2.2) | (0.0-1.9) | (0.0-2.6) |
| NP57 ^+^ neutrophils | 5.7 | 6.5 | 6.2 | 10.1 | 4.0 | 6,0 | 5.7 | 2.9 |
|  | (0.6-9.9) | (2.9-9.9) | (1.9-13.6) | (4.0-12.8) | (0.9-7.2) | (2.8-12.8) | (1.8-12.8) | (0.2-7.7) |
| % E-selectin | 0.0 | 0.0 | 0.0 | 0.0 | 0.0 | 0.0 | 0.0 | 2.9 |
| positive vessels | (0.0-3.6) | (0.0-0.0) | (0.0-6.5) | (0.0-0.0) | (0.0-0.0) | (0.0-5.9) | (0.0-1.7) | (0.0-7.7) |

**TABLE E2. ACUTE SMOKING EFFECTS IN OLD GROUPS**

**A. Neutrophil activation markers measured in blood by flow cytometry 2 hours after smoking**

|  | Change with smoking | | |
| --- | --- | --- | --- |
|  | Healthy controls | COPD | *p-value†* |
|  | *(n=24)* | *(n=13)* |  |
| CD16^+^ Neutrophils | 3.4 (0.4-10.2)* | 3.4 (0.3-6.4)* | NS |
| CD16^-^ Eosinophils | -1.8 (-3.0;-1.1)* | -2.9 (-5.1;-1.6)* | 0.049 |
|  |  |  |  |
| CD11b (Mac-1) | -6.5 (-19.8;-1.3) | 8.4 (-53.5;82.5) | NS |
| CD32 (FcγRII) | -6.5 (-19.8;-1.3)* | -12.4 (-20.8;-2.5)* | NS |
| CD54 (Icam-1) | -0.8 (-2.2;0.4) | -1.0 (-1.9;-0.1)* | NS |
| CD181/CXCR1 (IL-8 receptor) | -3.2 (-21.6;32.9) | -6.7 (-27.9;5.4) | NS |
| CD182/CXCR2 (IL-8 receptor) | -2.0 (-16.6;24.1) | -3.9 (-19.6;2.2) | NS |
| A17 (active FcγRII) | 3.1 (-8.5;30.8) | 7.0 (-13.1;32.9) | NS |
| A27 (active FcγRII) | 0.6 (-15.2;16.6) | 9.1 (-3.0;47.9) | NS |

Values are expressed as median change (T_3_-T_0_) in fluorescence intensity (MFI) with interquartile ranges (IQR), two hours after smoking. * Significant response to cigarette smoke within the group (Wilcoxon signed-rank tests, p<0.05). † p-values for differences in responses to cigarette smoke between susceptible and non-susceptible subjects (Mann-Whitney U tests, NS = not significant).

**B. Cytokines measured in blood 2 hours after smoking**

|  | Change with smoking | | |
| --- | --- | --- | --- |
|  | Healthy controls | COPD | *p-value†* |
|  | *(n=27)* | *(n=13)* |  |
| IL-1β | 0.00 (0.00;0.00) | 0.00 (0.00;0.00) | NS |
| IL-6 | -0.48 (-1.87;0.19)* | 0.10 (-0.49;0.95) | 0.081 |
| IL-8 | -0.62 (-1.34;0.21)* | 0.37 (-1.09;2.24) | 0.091 |
| GM-CSF | 0.00 (0.00;0.00) | 0.00 (0.00;0.00) | NS |
| TNFα | -0.71 (-1.20;0.00)* | -0.45 (-0.82;0.14) | NS |
| IFNγ | -0.37 (-1.58;0.58) | 0.50 (0.00;1.12) | NS |
| IL-2 | 0.00 (0.00;0.84) | 0.00 (-0.70;0.07) | NS |
| IL-4 | 0.00 (0.00;0.00) | 0.00 (-1.24;0.00) | NS |
| IL-5 | 0.00 (-0.10;0.09) | 0.00 (0.00;0.06) | NS |
| IL-7 | 0.00 (-0.28;1.30) | 1.21 (-0.85;4.58) | NS |
| IL-10 | 0.00 (-2.98;0.00) | -3.32 (-4.13;0.00)* | NS |
| IL-12p70 | 0.00 (-0.26;0.00) | 0.00 (-0.08;0.20) | NS |
| IL-13 | 0.00 (-1.58;0.87) | 0.00 (-1.15;1.65) | NS |

Values are expressed as median change (T_3_-T_0_) in cytokine concentration (pg/ml) with interquartile ranges (IQR), two hours after smoking. * Significant response to cigarette smoke within the group (Wilcoxon signed-rank tests, p<0.05). † p-values for differences in responses to cigarette smoke between susceptible and non-susceptible subjects (Mann-Whitney U tests, NS = not significant).

**C. Inflammatory cells in bronchial biopsies 24 hours after smoking**

|  | Change with smoking | | |
| --- | --- | --- | --- |
|  | Healthy controls | COPD | *p-value†* |
|  | *(n=20)* | *(n=12)* |  |
| *Submucosal* |  |  |  |
| CD3^+^ T-cells | -0.6 (-15.2;10.7) | -0.6 (-11.5;19.6) | NS |
| CD4^+^ T-cells | 1.2 (-3.5;5.2) | 1.3 (-0.7;3.2) | NS |
| CD8^+^ T-cells | 7.9 (-3.1;19.8)* | 1.7 (-4.3;13.9) | NS |
| FOXP3^+^ T-cells | 0.0 (-0.7;0.9) | 0.0 (0.0;2.6) | NS |
| CD68^+^ macrophages | 0.1 (-8.3;5.6) | -1.3 (-5.6;0.8) | NS |
| AA1^+^ mast cells | 0.7 (-2.7;3.1) | 0.0 (-1.5;2.4) | NS |
| EPX^+^ eosinophils | 0.41 (0.0;1.6) | 0.0 (-1.5;2.3) | NS |
| NP57 ^+^ neutrophils | 2.4 (-0.5;7.4)* | -2.4 (-7.3;1.7) | 0.027 |
| % E-selectin pos. vessels | 0.0 (0.0-1.1) | 1.2 (0.0;3.4)* | NS |

Values are expressed as median change(T_24_-T_0_) in cell counts with interquartile ranges (IQR), 24 hours after smoking. Inflammatory cells are expressed as cell counts / 0.1mm^2^. * Significant response to cigarette smoke within the group (Wilcoxon signed-rank tests, p<0.05). † p-values for differences in responses to cigarette smoke between susceptible and non-susceptible subjects (Mann-Whitney U tests, NS = not significant).

**REFERENCES**

(1) Koenderman L, Kanters D, Maesen B, Raaijmakers J, Lammers JW, de Kruif J, Logtenberg T. Monitoring of neutrophil priming in whole blood by antibodies isolated from a synthetic phage antibody library. *J Leukoc Biol* 2000;68:58-64.

(2) ten Hacken NH, Aleva RM, Oosterhoff Y, Smith M, Kraan J, Postma DS, Timens W. Submucosa 1.0 x 0.1 mm in size is sufficient to count inflammatory cell numbers in human airway biopsy specimens. *Mod Pathol* 1998;11:292-294.

(3) Broekema M, ten Hacken NH, Volbeda F, Lodewijk ME, Hylkema MN, Postma DS, Timens W. Airway epithelial changes in smokers but not in ex-smokers with asthma. *Am J Respir Crit Care Med* 2009;180:1170-1178.

(4) Broekema M, Timens W, Vonk JM, Volbeda F, Lodewijk ME, Hylkema MN, Ten Hacken NH, Postma DS. Persisting remodeling and less airway wall eosinophil activation in complete remission of asthma. *Am J Respir Crit Care Med* 2011;183:310-316.
